# Transcriptional and post-transcriptional responses to amyloid-**β** in cerebral amyloid angiopathy

**DOI:** 10.1177/0271678X251366082

**Published:** 2025-08-06

**Authors:** Barend W Florijn, Niek A Verwey, Ellis S van Etten, Helga E de Vries, Marieke JH Wermer

**Affiliations:** 1 Department of Neurology, 4501 Leiden University Medical Center, Leiden, the Netherlands; 2 Einthoven Laboratory for Vascular and Regenerative Medicine, Leiden University Medical Center, Leiden, the Netherlands; 3 Neuroscience Campus, VU University, Amsterdam, The Netherlands; 4 Neurology Department, Memory Clinic Geriatric Department, 4480 Medisch Centrum Leeuwarden, The Netherlands; 5 Department of Molecular Cell Biology and Immunology, Amsterdam UMC location Vrije Universiteit Amsterdam, Amsterdam, The Netherlands; 6 Department of Neurology, University Medical Center Groningen, Groningen, the Netherlands

**Keywords:** Cerebral amyloid angiopathy, amyloid-beta, neurovascular unit, transcripts, microRNA

## Abstract

Cerebral amyloid angiopathy (CAA) is a common age-related small vessel disease characterized by amyloid-beta (Aβ) accumulation in cortical and leptomeningeal blood vessel walls. Reduced Aβ clearance in the vasculature elevates the risk of CAA, while increasing evidence indicates that enhanced Aβ production in neurons also contributes. The impact of Aβ on the diverse cell types of the neurovascular unit (NVU)—including endothelial cells (ECs), pericytes, neurons, vascular smooth muscle cells (VSMCs), and astrocytes—remains unclear. This narrative review proposes that Aβ accumulation in NVUs during CAA drives a transcriptional response that reduces Aβ clearance while activating a neuron-specific post- transcriptional response that enhances Aβ production. Specifically, Aβ in NVUs was found to initiate a transcriptional cascade that destabilizes endothelial cells, increases blood-brain barrier permeability, and damages pericytes, ultimately inducing inflammatory and dysfunctional changes in VSMCs. These changes cause mitochondrial dysfunction and TGFβ deregulation in neurons, activating profibrotic mechanisms. Post-transcriptional regulation by microRNA networks in neurons affects Aβ processing by controlling the balance between amyloidogenic and non-amyloidogenic pathways through BACE1 and ADAM10 expression respectively. This review improves our understanding of Aβ accumulation in neurovascular units in CAA, potentially leading to better diagnostics, early biomarkers, and tools for prognosis and treatment.

## Introduction

CAA is a highly prevalent age-related cerebral small vessel disease^1^ and primarily characterized by Aβ-40 accumulation.^2^ Dutch-type cerebral amyloid angiopathy (Dutch-type CAA) is a hereditary form of CAA, caused by a specific genetic mutation in the amyloid precursor protein (APP) gene that promotes Aβ-42 deposition.^3^ Aβ42 predominantly deposits in capillaries, contributing to the formation of neuritic plaques.^4^ Moreover, it is proposed that Aβ42 initiates vascular Aβ accumulation by seeding in the vessel walls, which is subsequently exacerbated by Aβ40 deposition.^5^ In contrast, Aβ40 dominates in leptomeningeal and cortical artery walls, while in capillaries, the Aβ40:Aβ42 ratio is similar to plaques and lower than in arteries.^6^ This distribution could reflect differences in Aβ clearance, as the more soluble Aβ40 preferentially diffuses along cortical drainage pathways rather than through basal ganglia perforating arterioles. This is due to the brain’s interstitial fluid being primarily cleared along perivascular routes that follow cortical and leptomeningeal arteries.^7^ Although basal ganglia atrophy is observed in patients with CAA, it is not attributed to local Aβ deposition; instead, it likely represents a broader pattern of injury shared across brain regions.^8^ However, deep hemorrhages are identified in a minority of patients with iatrogenic CAA (a nonhereditary form occurring under age 55) but are likely due to a distinct pathophysiological mechanism—specifically, prion-like Aβ seeding—rather than local Aβ deposition.^9^

A definitive diagnosis of CAA can only be made post-mortem (unless it concerns the hereditary D-CAA form). During life, the diagnosis of probable or possible CAA relies on clinical symptoms in combination with MRI imaging, as defined by the Boston criteria version 2.0.^10^ These criteria include the use of both hemorrhagic imaging markers such as intracerebral hemorrhage (ICH), cerebral microbleeds,^11^ cortical superficial siderosis^12^ convexity subarachnoid hemorrhage, and non-hemorrhagic markers such as white matter hyperintensities in a spot like pattern and enlarged perivascular spaces in the centrum semi-ovale.^13^ Nonetheless, detecting early-stage CAA remains difficult due to the limited neuropathological correlation with MRI imaging (1). Besides, current radiological markers primarily capture irreversible vascular damage but fail to represent the underlying, potentially reversible processes. Furthermore, the Boston 2.0 criteria are less effective in diagnosing CAA in asymptomatic individuals.^14^ This underscores the need for better biomarkers that represent the (early) cellular response to Aβ accumulation in cortical and leptomeningeal vessel walls. We previously demonstrated in the setting of stroke that particularly non-coding RNA biomarkers are early biomarkers that could improve and accelerate diagnostics.^15^ Likewise, biomarkers that reflect early Aβ accumulation or cerebrovascular dysfunction could aid in developing staging tools to guide prognosis.

Aβ is a 4 kDa fragment of the APP, a larger precursor molecule mainly produced by neurons and vascular cells. Factors leading to Aβ accumulation in human cerebral microvessels include (i) impaired clearance mechanisms and (ii) increased production.^16^ Lacking conventional lymphatic vessels, the brain normally clears solutes via specialized pathways—the perivascular and paravascular spaces^17^—collectively forming the glymphatic system.^18^ This system clears Aβ when CSF flows from arterial to venous paravascular spaces, draining into deep cervical lymphatics. Normally the clearance of Aβ within the mouse brain is driven by vasomotion and characterized by low-frequency arteriolar oscillations.^19^ It is currently hypothesized that this process in mice involves NVUs, where neuronal activity regulates these spontaneous arteriolar oscillations.^20^ In humans this is driven by interactions between ECs and VSMCs in arterioles,^21^ as well as between ECs and pericytes in capillaries.^22^ However, in rTg-DI rats expressing the mutant human AβPP (which develop early-onset capillary Aβ deposition), microvascular Aβ and neuroinflammation obstruct glymphatic flow, divert CSF from the parenchyma, and reduce lymphatic drainage.^23^ Moreover, impaired Aβ clearance in patients with CAA activates ECs, causing pericyte degeneration, which in turn causes dysregulated cerebral blood flow, neurovascular uncoupling, and blood brain barrier (BBB) breakdown.^24^ This activates the amyloidogenic pathway in cultured human ECs and neurons, where elevated β-secretase (BACE1) expression increases APP cleavage, thereby leading to excessive Aβ production.^25^ Based on animal studies, this overwhelms the non-amyloidogenic pathway, involving alpha secretase (ADAM10) expression, which normally prevents Aβ formation by cleaving APP at the α-secretase site, preventing β- and γ-secretase processing.^26^ Collectively, the findings in mice therefore emphasize the contributions of cerebrovascular endothelial cells^27^ and neurons to Aβ production.^28^

In cerebral microvessels, the contribution of both ECs and neurons to Aβ production accelerates CAA progression. This was demonstrated by mechanistic studies where mice with endothelial-specific APP expression (causing elevated blood Aβ levels) are crossed with those expressing neuronal APP.^27^ Particularly endothelial BACE1 expression enhances APP cleavage, causing Aβ toxicity by reducing endothelial nitric oxide synthase (eNOS) activity.^29^ This impacts endothelial tight junction protein expression,^30^ especially in patients with CAA.^31^

In relation to neurons, autopsies of brains from patients with AD have shown increased BACE1 expression, which is associated with neuronal degeneration and elevated Aβ levels in the cerebral cortex compared to age-matched controls.^32^ Therefore, the presence of endothelial BACE1 expression in patients with CAA^31^ and neuronal BACE1 expression in patients with Alzheimer’s disease (AD)^32^ underscores the necessity of maintaining a balance between endothelial and neuronal functions in both amyloidogenic and non-amyloidogenic pathways. This is especially relevant as these cell types differ in their susceptibility and transcriptional responses to damage, each displaying unique gene expression patterns.^33^ While transcriptional activation plays a crucial role in the cellular response to Aβ accumulation, it is now evident that part of this response in NVUs is also regulated at the post-transcriptional level. This proceeds via differential microRNA expression like we previously reviewed in ischemic stroke.^34^ Therefore, a key question is how the transcriptional but also post-transcriptional response to Aβ in NVUs and neurons in CAA is characterized.

## Goal of the review

To explain impaired Aβ clearance (within ECs, VSMCs and pericytes) and increased Aβ production (within astrocytes and neurons), we propose that Aβ accumulation in NVUs in the context of CAA promotes a transcriptional response that decreases Aβ clearance while it instigates a neuronal specific post-transcriptional response that elevates Aβ production. To that extent, this narrative review has two objectives: 1) to examine the transcriptional response to Aβ in endothelial cells, pericytes, and VSMCs of the NVU in CAA and 2) to explore how differential microRNA expression in neurons influences the balance between amyloidogenic and non-amyloidogenic pathways, thereby driving Aβ production. The review starts with an overview of the composition and function of the neurovascular unit (NVU). Then it is followed by a review of transcriptional changes in NVU cells that are linked with impaired Aβ clearance in CAA (objective 1). Moreover, while all cells within the NVU are capable of producing Aβ peptides due to APP expression in all NVU cell types, research on the expression of APP, BACE1, and ADAM10 and their influence on Aβ production have so far been limited to neurons.^35^ Therefore, objective 2 focuses on investigating the post-transcriptional regulation of APP, BACE1, and ADAM10 mRNA expression by microRNAs in neurons, drawing primarily from Alzheimer’s disease literature but of high interest for future CAA research. Together, these insights underscore how cellular responses to Aβ involve complex transcriptional and post-transcriptional mechanisms, including noncoding RNAs like microRNAs.

## Search strategy

This review is based on data obtained from searches conducted in November 2024 across PubMed, Embase, Web of Science, and Cochrane, as well as a review of references from relevant articles using the following targeted search terms. (“Cerebral Amyloid Angiopathy”[MeSH] OR “HCHWA-D”[All Fields] OR (“cerebral”[All Fields] AND “amyloid”[All Fields] AND “angiopathy”[All Fields]) OR “cerebral amyloid angiopathies”[tw] OR “cerebral amyloid angiopathy”[title/abstract] OR “Cerebral Amyloid Angiopathy, Familial”[Mesh] OR “Autosomal Dominant Cerebrovascular Amyloidosis”[tw] OR “HCHWA”[tw] OR “hchwad”[tw] OR “Hereditary Cerebral Hemorrhage With Amyloidosis”[tw] OR “Hereditary Cerebral Haemorrhage With Amyloidosis”[tw] OR “Autosomal Dominant Cerebrovascular Amyloidosis”[title/abstract] OR “Icelandic Type Amyloidosis”[title/abstract] OR “Icelandic Type Cerebroarterial Amyloidosis”[title/abstract] OR ((“CAA”[tw] OR “dcaa”[tw] OR “d caa”[tw]) AND (“angiopathy”[tw] OR “angiopathies”[tw] OR “amyloid”[tw] OR “amyloid”[tw])) OR* (“cerebral”[ti] AND “amyloid angiopath”[ti]))* AND (“Neurovascular Unit”[All Fields] OR “Endothelial Cells”[MeSH] OR “Muscle, Smooth, Vascular”[Mesh] OR “Pericytes”[MeSH] OR “Neurons”[MeSH] OR “Vascular Smooth Muscle”[tw] OR “Neurovascular Units”[tw] OR “Endothelial Cells”[tw] OR “Endothelial Progenitor Cells”[tw] OR “Hemangioblasts”[tw] OR “Podosomes”[tw] OR “Pericytes”[tw] OR “Rouget Cells”[tw] OR “Neurons”[tw] OR “Nerve Cells”[tw] OR “Adrenergic Fibers”[tw] OR “Cholinergic Fibers”[tw] OR “Dendrites”[tw] OR “Axons”[tw] OR “Purkinje Cells”[tw] OR “Pyramidal Cells”[tw] OR “Interneurons”[tw] OR “Medium Spiny Neurons”[tw] OR “Mirror Neurons”[tw] OR “Neuropil”[tw] OR “Nissl Bodies”[tw] OR “Place Cells”[tw] OR “Retinal Bipolar Cells”[tw] OR “Sensory Receptor Cells”[tw] OR “Vestibular Hair Cells”[tw]) AND (“Transcriptional Activation”[MeSH] OR “RNA Processing, Post-Transcriptional”[Mesh] OR “MicroRNAs”[MeSH] OR “Gene Activation”[tw] OR “Trans-Activation”[tw] OR “Transactivation”[tw] OR “Post Transcriptional RNA Processing”[tw] OR “RNA Editing”[tw] OR “RNA Methylation”[tw] OR “RNA Splicing”[tw] OR “Alternative Splicing”[tw] OR “MicroRNAs”[tw] OR “miRNA”[tw] OR “miRNAs”[tw] OR “Small Temporal RNA”[tw] OR “stRNA”[tw]). Articles published between 1999 and June 2025 were included if they addressed transcriptional or post-transcriptional responses to amyloid-β within neurovascular units in the context of cerebral amyloid angiopathy. The final reference list was chosen based on their originality and relevance. Only original research articles published in English were included.

## NVUs regulate neurovascular coupling in response to neuronal activation

**Figure 1.**
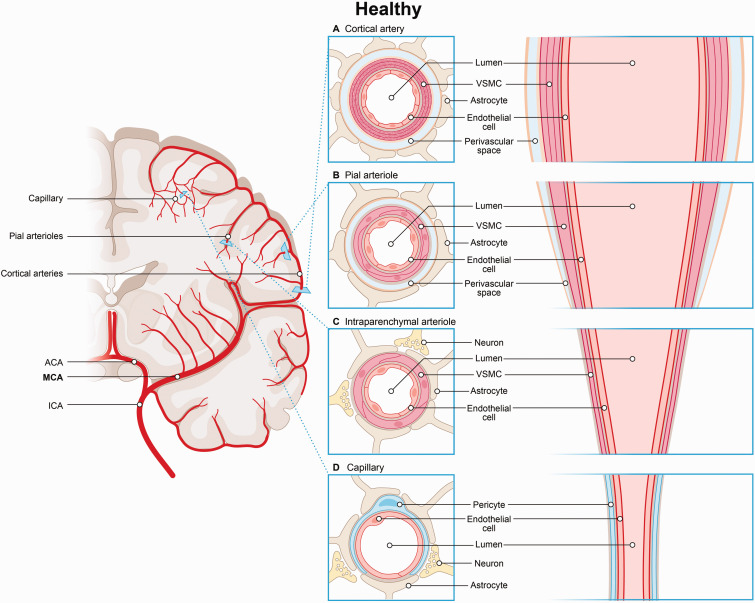
Segmental variation of the neurovascular unit. (a) In arteries, endothelial cells are surrounded by multiple layers of VSMCs and astrocytic end-feet, which support neurovascular coupling and maintain the integrity of the blood-brain barrier. (b) Larger arterioles have a single layer of VSMCs. Here the basement membranes of endothelial cells and VSMCs fuse but remain separated from the astrocytic basement membrane by the perivascular space. (c) In smaller arterioles, the vascular basement membrane merges with the astrocytic end-feet basement membrane, resulting in the loss of the perivascular space and (d) In capillaries, pericytes together with endothelial cells form the capillary wall and regulate cerebral blood flow.

A complex network of arteries, arterioles, and capillaries in the brain ensures the delivery of oxygen and glucose to neurons, thereby maintaining cerebral blood flow (CBF). NVUs regulate this process by adjusting vascular tone in response to neuronal activation, involving ECs and VSMCs in arteries and arterioles, and endothelial-pericyte interactions in capillaries (Figure 1).^22^ In arteries, endothelial cells are surrounded by multiple layers of VSMCs and astrocytic end-feet, which support neurovascular coupling and uphold blood-brain barrier integrity (Figure 1a).^22^ Arterioles have a single layer of VSMCs, with their basement membrane fusing with that of the endothelial cells, although both remain separated from the astrocytic basement membrane by the perivascular space (Figure 1b and c).^36^ In smaller arterioles, the vascular basement membrane merges with the basement membrane surrounding astrocytic end-feet, leading to the disappearance of the perivascular space (Figure 1c). In capillaries, VSMCs are replaced by pericytes, which, together with endothelial cells, form the capillary wall and share a common basement membrane (Figure 1d).^22^ Pericytes, embedded within the endothelial basement membrane, take over the regulatory roles of VSMCs at the capillary level, allowing them to control cerebral blood flow and maintain cerebrovascular integrity.^37^ As key components of the neurovascular unit in capillaries, pericytes are crucial for regulating blood-brain barrier (BBB) permeability, CBF, and Aβ clearance.^38^

Advances in single-cell/nuclear RNA sequencing have confirmed significant segmental diversity in the cerebral microvasculature,^36^ thereby revealing gene expression gradients along the arteriovenous axis that enable functional specialization.^39^ In ECs, this includes distinct transcription factor (TF) expression in arteries but transporter expression in capillaries and veins for perivascular clearance.^39^ Additionally, single-cell RNA-seq studies have revealed distinct gene expression patterns in VSMCs that are not present in capillary pericytes.^39^ This emphasizes a gradual transition from pericytes to VSMCs, characterized by the loss of pericyte-specific transcripts and the acquisition of VSMC markers.^40^ These findings highlight that as arteries branch into arterioles and capillaries, NVUs adapt their cellular composition to ensure consistent CBF regulation and thereby cerebrovascular clearance throughout the microvasculature. No single type of neurovascular unit is replicated at all levels of the cerebral vasculature. Instead, different NVUs with distinct molecular signatures support segmental variation.^36^

## The endothelial-pericyte transcriptional response to A**β** in NVUs

Mouse studies dedicated to the transcriptional response of cerebral endothelial cells to Aβ, often use transgenic mice with the Swedish APP mutation and exon 9 deletion in presenilin 1 (APPswe/PSEN1dE9). These mice display amyloid angiopathy alongside extensive parenchymal Aβ plaques beginning around 3 months-of-age, with a time-dependent increase in burden and extent. Therefore, caution is needed when extrapolating these results to the mechanistic understanding of CAA in human patients. Validation experiments in for example transgenic SwDI (TgSwDI) mouse models (with amyloid deposits primarily in the cerebral microvasculature at 6 months) is first needed. Nonetheless, early models of vascular Aβ accumulation in APPswe/PSEN1dE9 mice, demonstrate an endothelial-specific impact on cell cycle genes, with decreased G0 markers (CDK5, CDK5r1, CDK5r2) and increased G1 markers (CDK4, CDK6) exclusively in ECs. This indicates a shift to cell cycle reentry and enhanced susceptibility of ECs to cellular apoptosis.^41^ Moreover, an endothelial specific upregulation of genes involved in toll- like receptor (TLR) signaling and cytokine– cytokine receptor interaction (Tlr2, Tlr4, Irf7, Il1b), confirm loss of endothelial quiescence due to an innate immune response containing microglial activation.^41^ Consequently, microvessels in these mice show reduced endothelial expression of neurotransmitter receptors and calcium signaling transducers, indicating that the loss of endothelial quiescence disrupts neurovascular coupling and impairs Aβ clearance.^41^ Following Aβ accumulation, normal signaling between EC and neurons is compromised, thereby influencing CBF. This was particularly seen in a middle cerebral artery occlusion (MCAO) stroke model of 5xFAD mice (which overexpress APP and PSEN1, leading to severe amyloid angiopathy).^42^ After stroke, endothelial-specific reductions in genes like Sox18 and Cxcl12 altered cerebral EC-neuron communication.^43^ As such, this could highlight the importance of endothelial-neuronal signaling in regulating Aβ clearance.

Further studies in TgSwDI mice confirm that CAA promotes microglia activation, which form “cuff-like” structures around arterioles with Aβ deposition. Consequently, the transcriptional profile of cerebral microvessels from Tg-SwDI mice with Aβ deposition compared to control WT mice without amyloid, revealed enriched gene groups that relate to an enhanced immune response.^44^ Differentially expressed genes included genes related to lymphocyte co-stimulation (TNFSf4, CD274, CCL19), type 2 inflammatory response (XCL1, CD74, IL6) and upregulation of interferon-γ-related genes (CCL11, CCL20, CCL12, Tlr2). Interestingly, compared to young Tg-SwDI mice, aged Tg-SwDI mice showed increased CD40L expression (associated with B-cell immunity), suggesting an Aβ-driven proinflammatory phenotype with elements of both an adaptive and innate immune response, marked by strong macrophage/microglial activation.^44^ In older Tg-SwDI mice, this phenotype related to more cerebral microbleeds, indicating a role for age and an Aβ-dependent adaptive immune response in microbleed formation.^44^ In the capillaries of these mice, Aβ deposition was found to induce the growth of irregular capillaries, with fewer pericytes and altered cell morphology, leading to reduced endothelium-dependent functional hyperemia at 18 months.^45^ As such, also impaired pericyte function caused by Aβ deposition is contributing to an age-related decrease in Aβ clearance.

**Figure 2.**
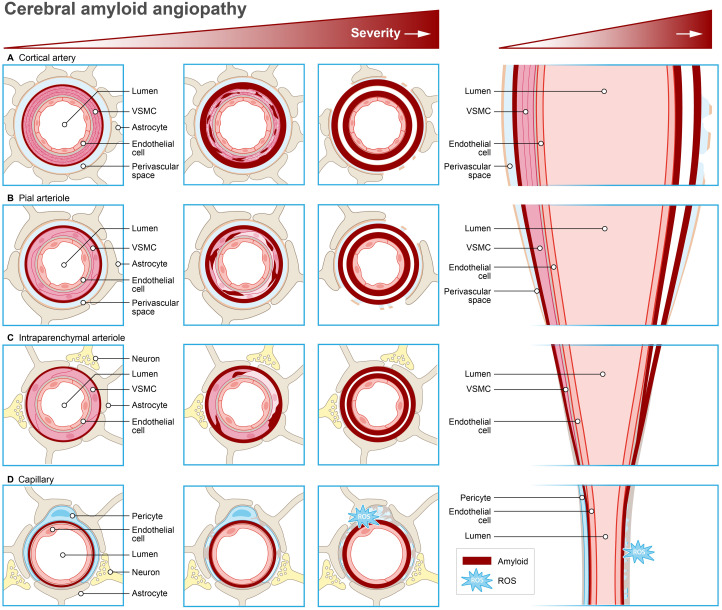
Transcriptional response to amyloid-β in neurovascular units in cerebral amyloid angiopathy. (a) In arteries, CAA is characterized by increased reactive astrocytes surrounding remodeled vessels and prominent concentric splitting of the vessel wall. (b) Early CAA formation is characterized by Aβ accumulation at the larger pial surface arterioles that follows the banding pattern of vascular smooth muscle cells, eventually reaching a confluent circumferential appearance. (c) In arterioles, Aβ is initially deposited within the outer basement membranes surrounding intact smooth muscle cells, sparing the basement membranes of the endothelium and (d) In capillaries, Aβ accumulation in the tunica intima (dark red line) disrupts endothelial quiescence, triggering reactive oxygen species (ROS) production and morphological damaged pericytes. Consequently, Aβ promotes capillary rarefaction, leading to irregular capillary growth with reduced pericyte coverage.

More interesting findings on the transcriptional response of cerebral ECs towards Aβ were identified in the Tg-ArcSwe mouse model. This model is based on the arc (activity-regulated cytoskeleton-associated protein) promoter, which drives the expression of the Swedish APP- and PSEN1 mutation in neurons. It is important to note that this is an AD mouse model with early Aβ pathology. Nevertheless, these mice display differential expression of genes involved in angiogenesis in cortical microvessels with CAA. Specifically, decreased levels of vascular endothelial growth factor receptor 2 (Vegfr2) mRNA was found alongside increased expression of angiopoietin 1 (Ang-1), angiopoietin 2 (Ang-2), and their Tie-2 receptor.^46^ These gene expression changes were found to correlate with morphologically damaged pericytes. However no significant alterations in pericyte coverage (which normally stabilize nearby endothelial cells) or vessel density were observed, suggesting CAA in capillaries contributes to global vasomotor dysfunction via capillary rarefaction (Figure 2d).^46^

Collectively, these findings indicate that endothelial quiescence is disrupted by innate and adaptive immune responses to Aβ accumulation in the tunica intima.^47^ This could potentially explain the findings from immunohistochemical studies of CAA-affected capillaries from human postmortem brains which show reduced tight-junction proteins (claudin-5, occludin) indicating BBB leakage.^48^ By driving capillary rarefaction, Aβ-activated endothelial cells and pericytes create an environment that make nearby vascular smooth muscle cells in the tunica media more vulnerable to Aβ40.^49^

## The transcriptional response of smooth muscle cells to A**β** in arteriolar neurovascular units

In two CAA and AD mouse models (Tg-SwDI and APP/PS1 mice) Aβ accumulation in VSMCs enhances the expression of serum response factor (SRF) and myocardin (MYOCD), two transcription factors that regulate VSMC differentiation.^50^ The resultant transcriptional switch appears specific to cerebral VSMCs, as MYOCD expression has not been detected in brain endothelial cells, astrocytes, or neurons.^50^ Consequently, the expression of low-density lipoprotein receptor-related protein 1 (LRP) — a receptor involved in Aβ clearance in VSMCs — is reduced, thereby transforming the VSMC phenotype into one that fails to clear Aβ.^50^ Similarly, in cortical and leptomeningeal blood vessels from individuals with AD, a decreased VSMC specific expression of neprilysin (a peptidase that participates in Aβ degradation and clearance) was seen, which coincided with increased Aβ deposition.^51^ One single study investigated the functional consequence of this altered transcriptional profile in cerebral VSMCs in response to Aβ accumulation. This study uncovered that CAA mice (particularly the Tg2576 strain which expresses human APP with the Swedish mutation that promotes Aβ40 formation) show disorganized VSMC layers within the vessel wall, prior to the onset of cell death.^52^ Consequently, VSMCs lose their ability to respond to both endothelial-dependent and independent vasodilatory stimuli thereby further compromising the perivascular clearance of Aβ40.^19,52–54^ Furthermore, disrupted VSMC layers contribute to vascular wall thickening,^55^ leading to the formation of microaneurysms and subsequent leakage.^56^ This process results in the replacement of VSMCs with connective tissue, ultimately replacing the entire artery wall (Figure 2a).^7^ Additionally, also the elevated expression of adhesion molecules by endothelial cells induces another phenotypic shift in VSMCs, particularly towards a pro-inflammatory state.^57^ This inflammatory state attracts microglial cells, increasing perivascular inflammation and NADPH oxidase-driven reactive oxygen species (ROS), both of which play critical roles in CAA-related vascular impairments.^58^ Interestingly, when inflammation was characterized in human cerebrovascular smooth muscle cells (HCSMCs) derived from postmortem Alzheimer’s disease patients (although after 14–21 days in culture), these cells also displayed elevated expression of complement pathway genes, including those from the classical complement pathway.^59^ As a result, VSMCs in autopsy tissue from patients with CAA (with various stages of CAA without evidence of microhemorrhage) exhibited reduced expression of smooth muscle cell actin, and reactive astrocytes were found surrounding the cerebral microvasculature (Figure 2a and b).^60^ These phenotypic switches in VSMCs could lead to two types of microvascular injury: microinfarcts or microbleeds, as revealed by serial sectioning of cerebral arterioles from patients with CAA.^61^ Interestingly in microbleeds, both Aβ and VSMCs are nearly absent at the bleeding site, while vessels linked to microinfarcts retained few intact VSMCs and consistently displayed vascular Aβ at the lesion core.^61^ These observations may be explained by impaired paravascular clearance of Aβ that leads to extensive Aβ build-up in vessels surrounding the ischemic area.^61^

## The transcriptional response of astrocyte endfeet to A**β**-induced arteriolar NVU dysfunction

Alterations in astrocyte morphology and function are known to occur in early stages of CAA pathology, as experiments with transgenic arctic β-amyloid (arc-Aβ) mice have shown.^62^ Transgenic arc-Aβ mice, expressing both Swedish (K670N/M671L) and Arctic (E693G) APP mutations, overproduce aggregation prone Aβ42, leading to significant vascular Aβ pathology.^62^ Advanced CAA in these mice led to reduced GLUT1 expression in ex vivo astrocytes, resulting in impaired lactate release, suggesting deficient glucose-to-lactate conversion—an energy source for neurons during stimulation.^63^ This reduction triggers a retraction of astrocyte endfeet (due to decreased β-dystroglycan expression), cellular swelling and neurovascular uncoupling.^63^ Moreover, studies in transgenic mice expressing the human APP, have confirmed that Aβ disrupts the normal structure of astrocyte endfeet around endothelial cells.^64^ Consequently, there is a loss of GFAP staining around blood vessels with excessive amyloid deposition.^64^ This is particularly seen in older APP mice in which there is a reduced presence of astrocytes around blood vessels, suggesting that the structural barrier created by astrocytes—the glia limitans—becomes compromised as the disease progresses.^65^ Interestingly, our group demonstrated that in human patients with presymptomatic D-CAA, GFAP levels in cerebrospinal fluid (CSF) act as an early biomarker for CAA, rising years before symptoms appear.^66^ In contrast, in symptomatic D-CAA patients, GFAP levels in both serum and CSF reflect advanced CAA.^66^

Next to its protective barrier functions, astrocytes have both pro-neuroinflammatory and anti-neuroinflammatory roles. Aβ accumulation triggers neuroinflammation via reactive astrogliosis, particularly around arterioles with CAA pathology. This was demonstrated in studies using the Tg-FDD mouse model of familial Danish dementia with early-stage CAA. Histological analysis of the cortex, hippocampus, and cerebellum has revealed significant perivascular astrogliosis, but not microgliosis, compared to WT mice which associated with neuronal death.^67^ RNA-Seq analysis of Tg-FDD mouse brains showed dysregulation of immune and lipid-related genes, including upregulation of TREM2, a key microglial receptor with a role in immune regulation and AD.^67^ These findings could suggest that astrocyte-driven neuroinflammation and vascular dysfunction may precede microglial activation thereby contributing to early neuronal injury in CAA. Interestingly, chronic cortical iron deposits in post-mortem tissue from patients with definite CAA, also associates with astrogliosis and iron presence in reactive astrocytes, although here activated microglia and macrophages were seen.^68^ Therefore, further research is required to characterize the transcriptional mechanisms driving the astrocyte response during astrogliosis.

## Transcriptional activation in neurons triggered by A**β**-induced neurovascular unit dysfunction

In a comprehensive transcriptome analysis of human post-mortem brain tissue from Dutch-type CAA patients, top down-regulated genes were involved in cellular aerobic respiration, including ATP synthesis and carbon metabolism indicating mitochondrial dysfunction.^69^ These differentially expressed genes were linked to the upregulation of endothelial leukocyte recruitment genes, including CCL2, CXCL2, and CSF1. Interestingly, genes involved in extracellular matrix (ECM)–receptor interaction (CD44 and ECM proteoglycans) were also upregulated, together with an increase in genes involved in transforming growth factor-beta (TGFβ) signaling (FN1, SERPINE1, TIMP-1). These findings, unaffected by post-mortem delay or loss of RNA quality, indicate that mitochondrial dysfunction and inflammation in endothelial cells drive the upregulation of ECM-related pathways and pro-fibrotic mechanisms in neurons. Consequently, CAA remodels the extracellular environment, disrupting ECM-receptor interactions and impairing the binding of ECM components like laminin, collagen, and fibronectin to neuronal receptors.^69^ Interestingly, in cerebral organoids cultured up to over 100 days and derived from fibroblasts from D-CAA patients, Aβ accumulation coincides with similar TGFβ pathway de-regulation, as demonstrated by increased TGFBR1 expression and elevated levels of neuronal precursor genes SOX1, SOX2, and PAX6. Mechanistically, the study suggests that elevated APP expression during brain development in D-CAA, which regulates neurogenesis, proliferation, and division, may also play a key role in promoting enhanced neuronal differentiation and maturation.^70^ This could suggest early prenatal disease despite mid-life onset. However, it could be argued that the absence of a vasculature and of neurovascular units is a significant limitation of the cerebral organoid in vitro model.

## Variants within the 3′UTR of APP mRNA disrupts microRNA binding sites

MicroRNAs are 21-nucleotide-long non-coding RNAs that regulate the expression of target messenger RNAs (mRNAs) by complementary base pairing with the 3′-untranslated region (3′-UTR) of mRNAs resulting in mRNA destabilization or translational inhibition.^71^ Several microRNAs directly target the 3′UTR of APP mRNA which is conserved in humans, rats, and mice and contains microRNA responsive elements. Genetic variants within this 3′UTR of APP mRNA can disrupt existing microRNA binding sites or lead to the creation of abnormal microRNA binding sites. This could for instance elevate APP mRNA or protein expression, thereby increasing the risk of CAA. A striking example of this was observed in a genetic screening study of the 3′-UTR of the APP gene in patients with CAA, revealing a sequence variant (c. *331_*332del) in the APP 3′UTR.^72^ This variant was associated with increased APP expression in probable CAA, likely due to the abnormal binding of two microRNAs (miR-582-3p and miR-892b) to APP mRNA.^72^ Specifically, miR-892b normally reduced APP production, but this effect was blocked by the CAA-associated mutation, while the sequence variant allowed miR-582-3p to bind, resulting in increased APP production.^72^

## Loss of microRNA function in neurons promotes the amyloidogenic pathway by disrupting BACE1 inhibition

**Figure 3.**
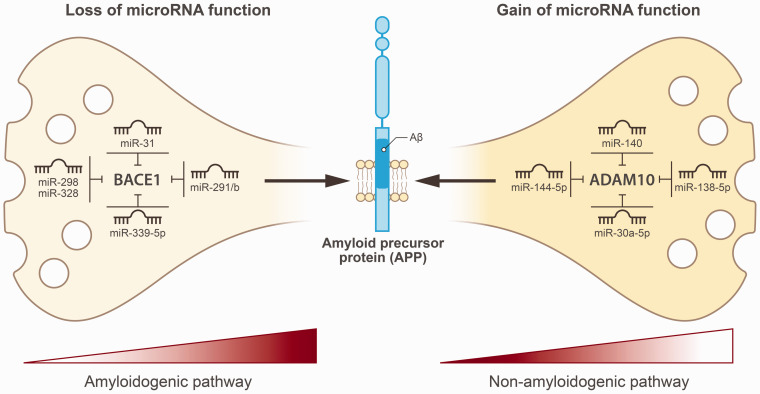
Post-transcriptional responses to amyloid-β in neurovascular units in cerebral amyloid angiopathy. Left panel depicts that loss of microRNA function in neurons promotes the amyloidogenic pathway by disrupting BACE1 inhibition. Right panel indicates that gain of microRNA function in neurons suppresses ADAM10 expression, thereby inactivating the non-amyloidogenic pathway and resulting in elevated Aβ production.

One important factor that associates with excessive Aβ production in neurons is increased BACE1 expression in the amyloidogenic pathway, which is particularly expressed in neurons. In neurons, certain neuronal microRNAs can simultaneously inhibit the expression of BACE1 and APP, thereby affecting Aβ processing and production. However, the loss of this microRNA function in neurons in an Alzheimer mouse model was found to promote the amyloidogenic pathway by removing the inhibitory regulation of BACE1. This effect was particularly observed with miR-31, a microRNA that was found to suppress both mRNA and protein levels of BACE1, as well as mRNA levels of the APP gene in hippocampal neurons from 17-month-old female AD triple-transgenic (3xTg-AD) mice.^73^ Functionally, this led to a significant improvement in memory deficits, reduced anxiety, and greater cognitive flexibility. Moreover, circulating miR-31 levels were also found to be decreased in the serum of patients with AD, thereby highlighting its potential as both a biomarker and a therapeutic target.^74^ More striking examples of cell-specific microRNA effects (Figure 3, Table 2) on BACE1 expression were seen in a study on miR-298, that showed it reduces APP and BACE1 expression in human astrocytes but not in differentiated neuroblastoma or microglia cells.^75^ This was explained by UTR-length variation between cells, which was found to prevent microRNA binding, thereby affecting its activity in specific cell types. However, it is worth noting that neuroblastoma cells were used, which have a deregulated cell cycle and display abnormal gene expression profiles. Nonetheless, also hippocampal neurons from APPSwe/PSEN1dE9 mice display decreased miR-298 expression levels, and decreased miR-328 expression levels, which were found to decline with age leading to increased BACE1 protein expression (and not BACE1 mRNA expression).^76^ Both microRNAs bind the 3′-UTR of BACE1 mRNA, although knocking down either microRNA individually did not significantly impact BACE1 expression, suggesting that miR-298 and miR-328 coordinately regulate its expression.^76^ More examples of BACE1 regulating microRNAs were found in neurons derived from postmortem AD brains (measured upon post-mortem intervals of 1.75 to 8 hours) in which miR-339-5p expression was decreased, resulting in higher BACE1 protein levels. In vitro studies in cultured primary human brain cells with target protectors against miR-339-5p binding sites on the BACE1 3′-UTR identified significantly increased BACE1 expression, suggesting BACE1 is a confirmed target of miR-339-5p.^77^ Interestingly, delivering miR-339-5p to mixed primary cultures of human fetal brain (HFB) cells (derived from the brain parenchyma of fetuses at 80–100 days of gestation) reduced both BACE1 protein expression but also Aβ levels.^77^ Lastly another microRNA example was found in human brain samples derived from the anterior temporal cortex. Herein the miR-29a/b-1 cluster was identified as suppressor of BACE1 protein expression (and not BACE1 mRNA expression suggesting no transcriptional regulation of mRNA expression).^78^ Also, the loss of function of the miR-29a/b-1 cluster in brain biopsies from patients with AD significantly elevated BACE1 protein levels, which were not region specific.^78^

## Post-transcriptional control of ADAM10 in the non-amyloidogenic pathway

Circulating microRNAs have shown potential as disease biomarkers, especially those targeting ADAM10 expression. In a small study of 21 patients with AD and 17 healthy controls, levels of miR-144-5p, miR-221, and miR-374 were reduced in patients with AD.^79^ Of those, miR-144-5p had the highest AUC for the detection of Alzheimer’s disease with a 66.7% sensitivity and 76.5% specificity.^79^ MicroRNA studies in post-mortem brain tissue can also provide insights into cellular changes associated with advanced disease stages. However, as post-mortem tissue reflects late-stage alterations, microRNA expression may differ from levels seen during earlier disease stages. Still in post-mortem cerebellum and hippocampus tissues from 21 patients with Alzheimer’s disease, expression levels of miR-140 were significantly elevated.^80^ Given hippocampal cell diversity and neuronal loss in late-stage AD, it remains uncertain if early-stage AD or specific cell types also show high miR-140. Therefore, authors conducted validation experiments in neuroblastoma cells (SHSY5Y, CHP212). Treatment of these cells with a miR-140-5p mimic reduced ADAM10 promoter 3′UTR reporter activity, confirming ADAM10 as a target of microRNA-140-5p.^80^ Other microRNAs that regulate ADAM10 expression were identified in mice in which high expression levels of miR-30a-5p were found in the cortex and hippocampus of APP/PS1 mice.^81^ These high expression levels led to neuronal damage by suppressing ADAM10 expression, thereby increasing Aβ production. Knockdown of miR-30a-5p in APP/PS1 mice improved cognition, reduced Aβ buildup, and promoted the nonamyloidogenic pathway by upregulating ADAM10.^81^ Interestingly, another study showed that overexpression of miR-34a in mice within the hippocampus and prefrontal cortex accelerates cognitive decline. MicroRNA-34a was found to target ADAM10 expression, leading to reduced ADAM10 protein in various brain regions and increased intraneuronal Aβ accumulation.^82^ Likewise, also age-related increases in miR-138 in APP/PS1 mice lowered ADAM10 expression, leading to higher Aβ production and resulting in synaptic and cognitive deficits, while its suppression reversed these effects.^83^ Together, these data suggest that increased microRNA activity suppresses ADAM10 expression, inactivating the non-amyloidogenic pathway and resulting in elevated Aβ production (Figure 3, Table 2).

## Discussion

We conclude that the transcriptional response associated with impaired Aβ clearance and accumulation in NVUs, is characterized by an endothelial-specific effect on cell cycle genes, suggesting increased susceptibility to apoptosis (Figure 2). Furthermore, upregulation of genes involved in TLR signaling and cytokine–cytokine receptor interaction, confirm loss of endothelial quiescence due to an innate immune response. This degenerates the endothelial lining of microvessels and disrupts endothelial stability by impacting the actin cytoskeleton and cell membrane. This changing of the endothelial phenotype raises BBB permeability, thereby impacting angiogenesis-related genes that stimulate capillary rarefaction following morphological pericyte damage. Subsequently, nearby VSMCs are transformed into an inflammatory, de-differentiated, and hypocontractile state, marked by the activation of transcription factors SRF and MYOCD. As a result, VSMCs lose responsiveness to vasodilatory signals, further impairing perivascular clearance of Aβ, potentially leading to microinfarcts or microbleeds. Because astrocyte endfoot integrity around ECs is also diminished, a reduced astrocytic GLUT1 expression disrupts neurovascular coupling. In neurons, this leads to loss of aerobic respiration functions, including ATP synthesis and carbon metabolism, pinpointing mitochondrial dysfunction and TGFβ pathway deregulation. Although the occipital cortex is more severely affected in CAA compared to other brain regions,^84^ we were unable to identify studies that investigated regional variations in neuronal transcriptional changes among these cortical areas. Therefore, future studies are needed to investigate region-specific variations in neuronal activity that could drive differences in gene transcription. Furthermore, the translational impact of the findings in this review is still limited given the use of different rodent models and post-mortem brain samples in the reviewed studies.^85^ Therefore, more studies in standardized and widely validated rodent models of CAA are needed to ensure consistency and comparability across studies. In addition, longitudinal studies in animal models and human patients with CAA are needed to track disease progression in NVUs and to validate the aforementioned findings at different stages of CAA.

Regarding the post-transcriptional response to Aβ in neurons we conclude that differential microRNA expression across NVU cell types can influence the balance between amyloidogenic and non-amyloidogenic Aβ processing. However, most microRNA research is conducted on AD patients. Still, we found that BACE1 expression in the amyloidogenic pathway is regulated at the post-transcriptional level in astrocytes (by miR-298) and especially in neurons (by the miR-29a/b-1 cluster, miR-31, miR-298, miR-328, and miR-339-5p). Conversely, the non-amyloidogenic pathway is regulated primarily by neuronal microRNAs (e.g., miR-30a-5p, miR-34a, miR-138, miR-140, and miR-221). These findings could suggest that BACE1 and ADAM10, within the amyloidogenic and non-amyloidogenic pathways respectively, are controlled by multiple microRNAs, forming a complex regulatory network that impacts Aβ accumulation in neurons. By regulating APP expression levels, microRNAs can influence the amount of substrate available for cleavage which could shift the balance between amyloidogenic and non-amyloidogenic processing and thereby the production of Aβ. However, as previously stated, all cells within the neurovascular unit have the capacity to produce Aβ peptides given that APP is expressed by all NVU cells.^35^ Therefore, a significant limitation of all the transgenic rodent models discussed (Table 1) is that the resulting CAA pathology arises ‘solely from a neuronal source’.^35^ This could not accurately represent the true pathogenesis of CAA because it fails to capture the complexity of Aβ’s cellular origin. Furthermore, more studies are needed to compare data across species, to identify conserved mechanisms and to validate the relevance of findings from rodent models to patients with CAA and from AD to CAA. Another important limitation of the reviewed studies is that some microRNA expression data in validation experiments is based on neuroblastoma cell lines. This highlights the need for direct CAA-specific research for a more precise understanding of these processes.

**Table 1.** Transcriptional response of NVU cells in different rodent models of CAA.

Study ID	Mouse model	NVU cell type	Biological pathway	Transcriptional response
Deng et al. 2022^41^	APPswe/PSEN1dE9 mice	ECs	Phases of the cell cycleInnate immune response	G0: Cdk5, Cdk5r1, Cdk5r2 ↓G1: Cdk4, Cdk6 ↑Tlr2, Tlr4, Irf7, Il1 ↑
Li et al. 2018^43^	MCAO stroke model of 5xFAD mice	ECs	Endothelial-neuronal signaling	Sox18, Cxcl12 ↓
Situ et al. 2022^44^	Tg-SwDI mice	ECs	Lymphocyte co-stimulationType 2 immune responseInterferon-γ signalling	Tnfsf4, Cd274, Ccl19 ↑Xcl1, Cd74, Il6 ↑Ccl11, Ccl20, Ccl12, Tlr2 ↑
Skaaraas et al. 2021^46^	Tg-ArcSwe mice	ECs/pericytes	Angiogenesis	Vegfr2 ↓, Ang-1, Ang-2, Tie-2 ↑
Bell et al. 2009^50^	Tg-SwDI and APPSwe mice	VSMCs	Aβ clearance	Srf and myocardin ↑
Merlini et al. 2011^63^	Tg-APPArcSwe	Astrocytes	Neuronal glucose uptake and astrocyte function	Glut1, β-dystroglycan ↓

**Table 2.** Post-transcriptional responses of NVU cells in rodent models and human CAA-cell lines.

Study ID	Mouse model/human cell line	NVU cell type	Post-transcriptional response
Amyloidogenic pathway			
Barros-Viegas et al.^73^	3xTg-AD mice	Neurons	miR-31- Bace1 and App
Wang et al.^75^	Human cell line	Astrocytes	miR-298 - Bace1 and App
Boissonneault et al.^76^	APPSwe/PSEN1dE9	Neurons	miR-298/miR-328 - Bace 1
Long et al.^77^	Human cell line	Neurons	miR-339-5p - Bace 1
Non-amyloidogenic pathway			
Akhter R et al.^80^	Human cell line	Neurons	miR-140 - Adam10
Sun et al.^81^	APP/PS1 mice	Neurons	miR-30a-5p - Adam10
Sarkar S et al.^82^	miR-34a^+/−^ mice	Neurons	miR-34a - Adam10
Lu et al.^83^	APP/PS1 mice	Neurons	miR-138 - Adam10

Understanding how Aβ alters gene and microRNA expression in NVU cell types could help identify early biomarkers of disease progression. Moreover, pinpointing key regulators of NVU dysfunction in CAA could reveal novel targets driving disease mechanisms. For example, siRNAs or antisense oligonucleotides targeting VSMC-enriched transcripts like myocardin could be promising therapeutic approaches. Lastly, identifying key transcriptional and post-transcriptional regulators involved in NVU dysfunction could improve the relevance of animal and in vitro models for human disease and drug screening. Still, several therapeutic strategies show promising potential for addressing differentially expressed genes that contribute to the complex pathophysiology of CAA. One promising approach is targeting specific pathways in NVU cells, such as TGF-β signaling in neurons. Evidence from a recent study in adult male Tg-SwDI mice, which received stereotaxic injections of lentivirus targeting TGF-β signaling in the retrotrapezoid nucleus, demonstrated improved cognitive performance.^86^ Another potential strategy involves developing therapies for specific cell types, such as proinflammatory astrocytes, given that A1 reactive astrocytes have been linked to the early stages of CAA pathology.^67^ Finally, the recently completed randomized controlled BATMAN trial aims to evaluate the effects of minocycline on neuroinflammatory markers (IL-6, MCP-1, and IBA-1) in cerebrospinal fluid of patients with sporadic CAA and D-CAA.^87^

## References

[1] Jäkel L De Kort AM Klijn CJM , et al. Prevalence of cerebral amyloid angiopathy: a systematic review and meta-analysis. Alzheimers Dement 2022; 18: 10–28. 34057813 10.1002/alz.12366 PMC9290643

[2] Koemans EA Chhatwal JP van Veluw SJ , et al. Progression of cerebral amyloid angiopathy: a pathophysiological framework. Lancet Neurol 2023; 22: 632–642. 37236210 10.1016/S1474-4422(23)00114-X

[3] Zhang-Nunes SX Maat-Schieman ML van Duinen SG , et al. The cerebral beta-amyloid angiopathies: hereditary and sporadic. Brain Pathol 2006; 16: 30–39. 16612980 10.1111/j.1750-3639.2006.tb00559.x PMC8095991

[4] Attems J Lintner F Jellinger KA. Amyloid beta peptide 1-42 highly correlates with capillary cerebral amyloid angiopathy and alzheimer disease pathology. Acta Neuropathol 2004; 107: 283–291. 14986026 10.1007/s00401-004-0822-6

[5] De Kort AM Kuiperij HB Marques TM , et al. Decreased cerebrospinal fluid amyloid β 38, 40, 42, and 43 levels in sporadic and hereditary cerebral amyloid angiopathy. Ann Neurol 2023; 93: 1173–1186. 36707720 10.1002/ana.26610 PMC10238617

[6] Greenberg SM Bacskai BJ Hernandez-Guillamon M , et al. Cerebral amyloid angiopathy and alzheimer disease – one peptide, two pathways. Nat Rev Neurol 2020; 16: 30–42. 31827267 10.1038/s41582-019-0281-2 PMC7268202

[7] Keable A Fenna K Yuen HM , et al. Deposition of amyloid β in the walls of human leptomeningeal arteries in relation to perivascular drainage pathways in cerebral amyloid angiopathy. Biochim Biophys Acta 2016; 1862: 1037–1046. 26327684 10.1016/j.bbadis.2015.08.024 PMC4827375

[8] Fotiadis P Pasi M Charidimou A , et al. Decreased basal ganglia volume in cerebral amyloid angiopathy. J Stroke 2021; 23: 223–233. 34102757 10.5853/jos.2020.04280 PMC8189850

[9] Kaushik K van Etten ES Siegerink B , et al. Iatrogenic cerebral amyloid angiopathy post neurosurgery: frequency, clinical profile, radiological features, and outcome. Stroke 2023; 54: 1214–1223. 37035916 10.1161/STROKEAHA.122.041690 PMC10121246

[10] Charidimou A Boulouis G Frosch MP , et al. The boston criteria version 2.0 for cerebral amyloid angiopathy: a multicentre, retrospective, MRI-neuropathology diagnostic accuracy study. Lancet Neurol 2022; 21: 714–725. 35841910 10.1016/S1474-4422(22)00208-3 PMC9389452

[11] Greenberg SM Vernooij MW Cordonnier C , Microbleed Study Group et al. Cerebral microbleeds: a guide to detection and interpretation. Lancet Neurol 2009; 8: 165–174. 19161908 10.1016/S1474-4422(09)70013-4 PMC3414436

[12] Linn J Halpin A Demaerel P , et al. Prevalence of superficial siderosis in patients with cerebral amyloid angiopathy. Neurology 2010; 74: 1346–1350. 20421578 10.1212/WNL.0b013e3181dad605 PMC2875936

[13] Wardlaw JM Smith EE Biessels GJ , et al. Neuroimaging standards for research into small vessel disease and its contribution to ageing and neurodegeneration. Lancet Neurol 2013; 12: 822–838. 23867200 10.1016/S1474-4422(13)70124-8 PMC3714437

[14] Switzer A Charidimou A McCarter S , et al. Diagnostic value of the boston criteria v2.0 for cerebral amyloid angiopathy in individuals without hemorrhage: an MRI-neuropathological validation study (S15.009). Neurology 2023; 100: 3516.

[15] Florijn BW Leontien van der Bent M Nguyen TMT , et al. Non-coding RNAs versus protein biomarkers to diagnose and differentiate acute stroke: systematic review and meta-analysis. J Stroke Cerebrovasc Dis 2023; 32: 107388–20230929. 37778160 10.1016/j.jstrokecerebrovasdis.2023.107388

[16] Greenberg SM Charidimou A. Diagnosis of cerebral amyloid angiopathy: evolution of the boston criteria. Stroke 2018; 49: 491–497. 29335334 10.1161/STROKEAHA.117.016990 PMC5892842

[17] Iliff JJ Wang M Liao Y , et al. A paravascular pathway facilitates CSF flow through the brain parenchyma and the clearance of interstitial solutes, including amyloid β. Sci Transl Med 2012; 4: 147ra111. 10.1126/scitranslmed.3003748 PMC3551275 22896675

[18] Lui F Alcaide J Knowlton S , et al. Pathogenesis of cerebral amyloid angiopathy caused by chaotic glymphatics – mini-review. Front Neurosci 2023; 17: 1180237–20230411. 37113157 10.3389/fnins.2023.1180237 PMC10126375

[19] van Veluw SJ Hou SS Calvo-Rodriguez M , et al. Vasomotion as a driving force for paravascular clearance in the awake mouse brain. Neuron 2020; 105: 549–561.e5. 31810839 10.1016/j.neuron.2019.10.033 PMC7028316

[20] Mateo C Knutsen PM Tsai PS , et al. Entrainment of arteriole vasomotor fluctuations by neural activity is a basis of blood-oxygenation-level-dependent “resting-state” connectivity. Neuron 2017; 96: 936–948.e3. 29107517 10.1016/j.neuron.2017.10.012 PMC5851777

[21] McConnell HL Kersch CN Woltjer RL , et al. The translational significance of the neurovascular unit. J Biol Chem 2017; 292: 762–770. 27920202 10.1074/jbc.R116.760215 PMC5247651

[22] Iadecola C. The neurovascular unit coming of age: a journey through neurovascular coupling in health and disease. Neuron 2017; 96: 17–42. 28957666 10.1016/j.neuron.2017.07.030 PMC5657612

[23] Chen X Liu X Koundal S , et al. Cerebral amyloid angiopathy is associated with glymphatic transport reduction and time-delayed solute drainage along the neck arteries. Nat Aging 2022; 2: 214–223. 36199752 10.1038/s43587-022-00181-4 PMC9531841

[24] Hartz AM Bauer B Soldner EL , et al. Amyloid-β contributes to blood-brain barrier leakage in transgenic human amyloid precursor protein mice and in humans with cerebral amyloid angiopathy. Stroke 2012; 43: 514–523. 22116809 10.1161/STROKEAHA.111.627562 PMC5761312

[25] Zhang X Song W. The role of APP and BACE1 trafficking in APP processing and amyloid-β generation. Alzheimers Res Ther 2013; 5: 46. 24103387 10.1186/alzrt211 PMC3978418

[26] Kuhn PH Wang H Dislich B , et al. ADAM10 is the physiologically relevant, constitutive alpha-secretase of the amyloid precursor protein in primary neurons. EMBO J 2010; 29: 3020–3032. 20676056 10.1038/emboj.2010.167 PMC2944055

[27] Tachida Y Miura S Muto Y , et al. Endothelial expression of human amyloid precursor protein leads to amyloid β in the blood and induces cerebral amyloid angiopathy in knock-in mice. J Biol Chem 2022; 298: 101880–20220331. 35367207 10.1016/j.jbc.2022.101880 PMC9144051

[28] Herzig MC Winkler DT Burgermeister P , et al. Abeta is targeted to the vasculature in a mouse model of hereditary cerebral hemorrhage with amyloidosis. Nat Neurosci 2004; 7: 954–960. 15311281 10.1038/nn1302

[29] Zhou H Gao F Yang X , et al. Endothelial BACE1 impairs cerebral small vessels via tight junctions and eNOS. Circ Res 2022; 130: 1321–1341. 35382554 10.1161/CIRCRESAHA.121.320183

[30] Katusic ZS d’Uscio LV He T. Cerebrovascular endothelial dysfunction: Role of BACE1. Arterioscler Thromb Vasc Biol 2024; 44: 1737–1747. 38868939 10.1161/ATVBAHA.124.320798 PMC11269044

[31] Cheng X He P Yao H , et al. Occludin deficiency with BACE1 elevation in cerebral amyloid angiopathy. Neurology 2014; 82: 1707–1715. 24739782 10.1212/WNL.0000000000000403 PMC4032211

[32] Yang LB Lindholm K Yan R , et al. Elevated beta-secretase expression and enzymatic activity detected in sporadic alzheimer disease. Nat Med 2003; 9: 3–4. 12514700 10.1038/nm0103-3

[33] Rajput P Brookshier A Kothari S , et al. Differential vulnerability and response to injury among brain cell types comprising the neurovascular unit. J Neurosci 2024; 44 : 20240529. 10.1523/JNEUROSCI.1093-22.2024 PMC11140689 38548341

[34] Florijn BW Bijkerk R Kruyt ND , et al. Sex-specific MicroRNAs in neurovascular units in ischemic stroke. Int J Mol Sci 2021; 22: 20211102. 10.3390/ijms222111888 PMC8585074 34769320

[35] van Veluw SJ Benveniste H Bakker E , et al. Is CAA a perivascular brain clearance disease? A discussion of the evidence to date and outlook for future studies. Cell Mol Life Sci 2024; 81: 239–20240527. 38801464 10.1007/s00018-024-05277-1 PMC11130115

[36] Schaeffer S Iadecola C. Revisiting the neurovascular unit. Nat Neurosci 2021; 24: 1198–1209. 34354283 10.1038/s41593-021-00904-7 PMC9462551

[37] Hall CN Reynell C Gesslein B , et al. Capillary pericytes regulate cerebral blood flow in health and disease. Nature 2014; 508: 55–60. 24670647 10.1038/nature13165 PMC3976267

[38] Anwar MM Özkan E Gürsoy-Özdemir Y. The role of extracellular matrix alterations in mediating astrocyte damage and pericyte dysfunction in Alzheimer’s disease: a comprehensive review. Eur J Neurosci 2022; 56: 5453–5475. 34182602 10.1111/ejn.15372

[39] Vanlandewijck M He L Mäe MA , et al. A molecular atlas of cell types and zonation in the brain vasculature. Nature 2018; 554: 475–480. 29443965 10.1038/nature25739

[40] Saunders A Macosko EZ Wysoker A , et al. Molecular diversity and specializations among the cells of the adult mouse brain. Cell 2018; 174: 1015–1030.e16. 30096299 10.1016/j.cell.2018.07.028 PMC6447408

[41] Deng W Guo S van Veluw SJ , et al. Effects of cerebral amyloid angiopathy on the brain vasculome. Aging Cell 2022; 21: e13503. 35851991 10.1111/acel.13503 PMC9381891

[42] Forner S Kawauchi S Balderrama-Gutierrez G , et al. Systematic phenotyping and characterization of the 5xFAD mouse model of alzheimer’s disease. Sci Data 2021; 8: 270. 34654824 10.1038/s41597-021-01054-y PMC8519958

[43] Li Y Chang S Li W , et al. cxcl12-engineered endothelial progenitor cells enhance neurogenesis and angiogenesis after ischemic brain injury in mice. Stem Cell Res Ther 2018; 9: 139. 29751775 10.1186/s13287-018-0865-6 PMC5948880

[44] Situ M Citalan-Madrid AF Stamatovic SM , et al. Transcriptomic profile of blood-brain barrier remodeling in cerebral amyloid angiopathy. Front Cell Neurosci 2022; 16: 931247. 35813502 10.3389/fncel.2022.931247 PMC9257207

[45] Park L Koizumi K El Jamal S , et al. Age-dependent neurovascular dysfunction and damage in a mouse model of cerebral amyloid angiopathy. Stroke 2014; 45: 1815–1821. 24781082 10.1161/STROKEAHA.114.005179 PMC4284427

[46] Skaaraas G Melbye C Puchades MA , et al. Cerebral amyloid angiopathy in a mouse model of alzheimer’s disease associates with upregulated angiopoietin and downregulated hypoxia-inducible factor. J Alzheimers Dis 2021; 83: 1651–1663. 34459401 10.3233/JAD-210571 PMC8609707

[47] Carrano A Hoozemans JJ van der Vies SM , et al. Neuroinflammation and blood-brain barrier changes in capillary amyloid angiopathy. Neurodegener Dis 2012; 10: 329–331. 22301467 10.1159/000334916

[48] Freeze WM Bacskai BJ Frosch MP , et al. Blood-brain barrier leakage and microvascular lesions in cerebral amyloid angiopathy. Stroke 2019; 50: 328–335. 30661497 10.1161/STROKEAHA.118.023788 PMC6415745

[49] Charidimou A Boulouis G Gurol ME , et al. Emerging concepts in sporadic cerebral amyloid angiopathy. Brain 2017; 140: 1829–1850. 28334869 10.1093/brain/awx047 PMC6059159

[50] Bell RD Deane R Chow N , et al. SRF and myocardin regulate LRP-mediated amyloid-beta clearance in brain vascular cells. Nat Cell Biol 2009; 11: 143–153. 19098903 10.1038/ncb1819 PMC2654279

[51] Carpentier M Robitaille Y DesGroseillers L , et al. Declining expression of neprilysin in alzheimer disease vasculature: possible involvement in cerebral amyloid angiopathy. J Neuropathol Exp Neurol 2002; 61: 849–856. 12387451 10.1093/jnen/61.10.849

[52] Christie R Yamada M Moskowitz M , et al. Structural and functional disruption of vascular smooth muscle cells in a transgenic mouse model of amyloid angiopathy. Am J Pathol 2001; 158: 1065–1071. 11238054 10.1016/S0002-9440(10)64053-9 PMC1850363

[53] Shin HK Jones PB Garcia-Alloza M , et al. Age-dependent cerebrovascular dysfunction in a transgenic mouse model of cerebral amyloid angiopathy. Brain 2007; 130: 2310–2319. 17638859 10.1093/brain/awm156

[54] Dietrich HH Xiang C Han BH , et al. Soluble amyloid-beta, effect on cerebral arteriolar regulation and vascular cells. Mol Neurodegener 2010; 5: 15–20100413. 20388225 10.1186/1750-1326-5-15 PMC2873254

[55] Love S Chalmers K Ince P , et al. Development, appraisal, validation and implementation of a consensus protocol for the assessment of cerebral amyloid angiopathy in post-mortem brain tissue. Am J Neurodegener Dis 2014; 3: 19–32. 20140328. 24754000 PMC3986608

[56] Attems J Jellinger K Thal DR , et al. Review: sporadic cerebral amyloid angiopathy. Neuropathol Appl Neurobiol 2011; 37: 75–93. 20946241 10.1111/j.1365-2990.2010.01137.x

[57] Vromman A Trabelsi N Rouxel C , et al. β-amyloid context intensifies vascular smooth muscle cells induced inflammatory response and de-differentiation. Aging Cell 2013; 12: 358–369. 23425004 10.1111/acel.12056

[58] Han BH Zhou ML Abousaleh F , et al. Cerebrovascular dysfunction in amyloid precursor protein transgenic mice: contribution of soluble and insoluble amyloid-beta peptide, partial restoration via gamma-secretase inhibition. J Neurosci 2008; 28: 13542–13550. 19074028 10.1523/JNEUROSCI.4686-08.2008 PMC2626633

[59] Walker DG Dalsing-Hernandez JE Lue LF. Human postmortem brain-derived cerebrovascular smooth muscle cells express all genes of the classical complement pathway: a potential mechanism for vascular damage in cerebral amyloid angiopathy and alzheimer’s disease. Microvasc Res 2008; 75: 411–419. 18048067 10.1016/j.mvr.2007.10.004 PMC2774213

[60] Kozberg MG Yi I Freeze WM , et al. Blood-brain barrier leakage and perivascular inflammation in cerebral amyloid angiopathy. Brain Commun 2022; 4: fcac245. 36267331 10.1093/braincomms/fcac245 PMC9576155

[61] van Veluw SJ Scherlek AA Freeze WM , et al. Different microvascular alterations underlie microbleeds and microinfarcts. Ann Neurol 2019; 86: 279–292. 31152566 10.1002/ana.25512 PMC8722100

[62] Knobloch M Konietzko U Krebs DC , et al. Intracellular abeta and cognitive deficits precede beta-amyloid deposition in transgenic arcAbeta mice. Neurobiol Aging 2007; 28: 1297–1306. 16876915 10.1016/j.neurobiolaging.2006.06.019

[63] Merlini M Meyer EP Ulmann-Schuler A , et al. Vascular β-amyloid and early astrocyte alterations impair cerebrovascular function and cerebral metabolism in transgenic arcAβ mice. Acta Neuropathol 2011; 122: 293–311. 21688176 10.1007/s00401-011-0834-y PMC3168476

[64] Zago W Schroeter S Guido T , et al. Vascular alterations in PDAPP mice after anti-aβ immunotherapy: implications for amyloid-related imaging abnormalities. Alzheimers Dement 2013; 9: S105–115. 23583235 10.1016/j.jalz.2012.11.010

[65] Kim SH Ahn JH Yang H , et al. Cerebral amyloid angiopathy aggravates perivascular clearance impairment in an Alzheimer’s disease mouse model. Acta Neuropathol Commun 2020; 8: 181. 33153499 10.1186/s40478-020-01042-0 PMC7643327

[66] Rasing I Voigt S Koemans EA , et al. Serum and cerebrospinal fluid neurofilament light chain and glial fibrillary acid protein levels in early and advanced stages of cerebral amyloid angiopathy. Alzheimers Res Ther 2024; 16: 86. 38654326 10.1186/s13195-024-01457-0 PMC11036675

[67] Taylor X Cisternas P You Y , et al. A1 reactive astrocytes and a loss of TREM2 are associated with an early stage of pathology in a mouse model of cerebral amyloid angiopathy. J Neuroinflammation 2020; 17: 223. 32711525 10.1186/s12974-020-01900-7 PMC7382050

[68] Auger CA Perosa V Greenberg SM , et al. Cortical superficial siderosis is associated with reactive astrogliosis in cerebral amyloid angiopathy. J Neuroinflammation 2023; 20: 195–20230827. 37635208 10.1186/s12974-023-02872-0 PMC10463916

[69] Grand Moursel L van Roon-Mom WMC Kiełbasa SM , et al. Brain transcriptomic analysis of hereditary cerebral hemorrhage with amyloidosis-Dutch type. Front Aging Neurosci 2018; 10: 102. 29706885 10.3389/fnagi.2018.00102 PMC5908973

[70] Daoutsali E Pepers BA Stamatakis S , et al. Amyloid beta accumulations and enhanced neuronal differentiation in cerebral organoids of Dutch-type cerebral amyloid angiopathy patients. Front Aging Neurosci 2022; 14: 1048584. 36733499 10.3389/fnagi.2022.1048584 PMC9887998

[71] Esteller M. Non-coding RNAs in human disease. Nat Rev Genet 2011; 12: 861–874. 22094949 10.1038/nrg3074

[72] Nicolas G Wallon D Goupil C , et al. Mutation. in the 3′untranslated region of APP as a genetic determinant of cerebral amyloid angiopathy. Eur J Hum Genet 2016; 24: 92–98 25828868 10.1038/ejhg.2015.61 PMC4795229

[73] Barros-Viegas AT Carmona V Ferreiro E , et al. miRNA-31 improves cognition and abolishes amyloid-β pathology by targeting APP and BACE1 in an animal model of Alzheimer’s disease. Mol Ther Nucleic Acids 2020; 19: 1219–1236. 32069773 10.1016/j.omtn.2020.01.010 PMC7031139

[74] Dong H Li J Huang L , et al. Serum MicroRNA profiles serve as novel biomarkers for the diagnosis of Alzheimer’s disease. Dis Markers 2015; 2015: 625659. 26078483 10.1155/2015/625659 PMC4452867

[75] Wang R Lahiri DK. Effects of microRNA-298 on APP and BACE1 translation differ according to cell type and 3′-UTR variation. Sci Rep 2022; 12: 3074. 35197498 10.1038/s41598-022-05164-4 PMC8866491

[76] Boissonneault V Plante I Rivest S , et al. MicroRNA-298 and microRNA-328 regulate expression of mouse beta-amyloid precursor protein-converting enzyme 1. J Biol Chem 2009; 284: 1971–1981. 18986979 10.1074/jbc.M807530200 PMC2908704

[77] Long JM Ray B Lahiri DK. MicroRNA-339-5p down-regulates protein expression of β-site amyloid precursor protein-cleaving enzyme 1 (BACE1) in human primary brain cultures and is reduced in brain tissue specimens of alzheimer disease subjects. J Biol Chem 2014; 289: 5184–5198. 24352696 10.1074/jbc.M113.518241 PMC3931075

[78] Hébert SS Horré K Nicolaï L , et al. Loss of microRNA cluster miR-29a/b-1 in sporadic Alzheimer’s disease correlates with increased BACE1/beta-secretase expression. Proc Natl Acad Sci U S A 2008; 105: 6415–6420. 18434550 10.1073/pnas.0710263105 PMC2359789

[79] Manzine PR Pelucchi S Horst MA , et al. microRNA 221 targets ADAM10 mRNA and is downregulated in Alzheimer’s disease. J Alzheimers Dis 2018; 61: 113–123. 29036829 10.3233/JAD-170592

[80] Akhter R Shao Y Shaw M , et al. Regulation of ADAM10 by miR-140-5p and potential relevance for Alzheimer’s disease. Neurobiol Aging 2018; 63: 110–119. 29253717 10.1016/j.neurobiolaging.2017.11.007 PMC5800962

[81] Sun T Zhao K Liu M , et al. miR-30a-5p induces aβ production via inhibiting the nonamyloidogenic pathway in Alzheimer’s disease. Pharmacol Res 2022; 178: 106153. 35257899 10.1016/j.phrs.2022.106153

[82] Sarkar S Engler-Chiurazzi EB Cavendish JZ , et al. Over-expression of miR-34a induces rapid cognitive impairment and Alzheimer’s disease-like pathology. Brain Res 2019; 1721: 146327. 31295467 10.1016/j.brainres.2019.146327 PMC7014559

[83] Lu Y Tan L Wang X. Circular HDAC9/microRNA-138/sirtuin-1 pathway mediates synaptic and amyloid precursor protein processing deficits in Alzheimer’s disease. Neurosci Bull 2019; 35: 877–888. 30887246 10.1007/s12264-019-00361-0 PMC6754481

[84] Attems J Quass M Jellinger KA , et al. Topographical distribution of cerebral amyloid angiopathy and its effect on cognitive decline are influenced by Alzheimer disease pathology. J Neurol Sci 2007; 257: 49–55. 17306303 10.1016/j.jns.2007.01.013

[85] van Veluw SJ Benveniste H van Osch MJP. A translational approach towards understanding brain waste clearance in cerebral amyloid angiopathy. Eur Heart J 2024; 45: 1500–1502. 38289319 10.1093/eurheartj/ehae011

[86] El Hamamy A Iqbal Z Le NM , et al. Targeted TGF-βR2 silencing in the retrotrapezoid nucleus mitigates respiratory dysfunction and cognitive decline in a mouse model of cerebral amyloid angiopathy with and without stroke. Transl Stroke Res 2025; 16: 1272–1284. 39543011 10.1007/s12975-024-01306-0

[87] Voigt S Koemans EA Rasing I , et al. Minocycline for sporadic and hereditary cerebral amyloid angiopathy (BATMAN): study protocol for a placebo-controlled randomized double-blind trial. Trials 2023; 24: 378. 37277877 10.1186/s13063-023-07371-4 PMC10241553

